# Roth spots and retinal haemorrhages as initial presentations of chronic myeloid leukaemia

**DOI:** 10.1002/jha2.216

**Published:** 2021-05-29

**Authors:** Nkemdirim Jacob, Mike Leach, Deepak Tejwani, Mike Manson, Fraser Patrick

**Affiliations:** ^1^ Haematology Department Beatson West of Scotland Cancer Centre Glasgow UK; ^2^ Opthalmology Department Royal Alexandra Hospital Paisley Scotland; ^3^ Haematology Department Royal Alexandra Hospital Paisley Scotland


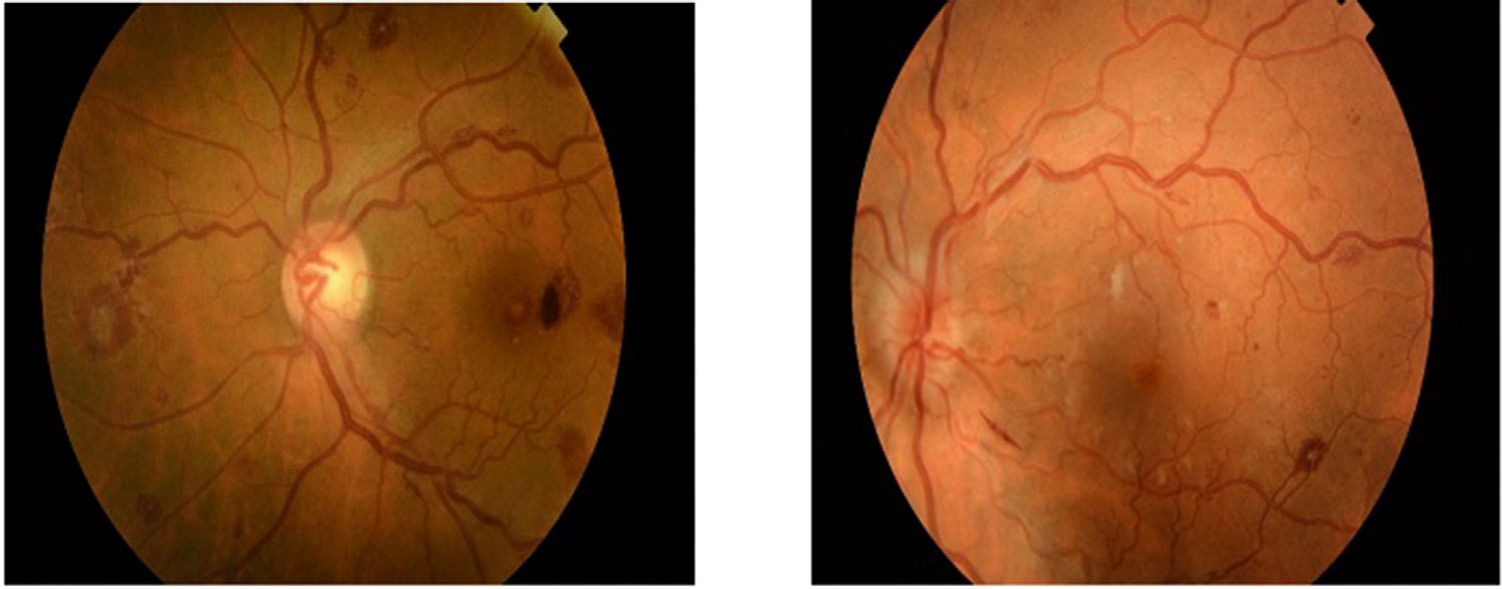


A 62‐year‐old woman with no significant past medical history was referred to the ophthalmologist on account of a 3‐week history of blurred vision. Examination revealed visual acuities of 6/36 and 6/18 for her right and left eye, respectively. Anterior segments and intraocular pressures were normal but she had bilateral multiple widespread retinal haemorrhages with Roth spots (image on the left above). Full blood count showed a significantly high white blood cell count (WBC) at 542 × 10^9^/L, haemoglobin (Hb) 110 g/L and platelets 256 × 10^9^/L and a blood film appearance in keeping with chronic myeloid leukaemia (CML). Her *BCR‐ABL1* mutation screen was positive and her bone marrow did not show an excess of blasts. Chronic‐phase CML was diagnosed and adequate treatment was instituted.

A 40‐year‐old man was seen by the opticians for a visual assessment on account of 2‐month history of blurring of central vision. His visual acuity was largely preserved. The opticians then referred him to the ophthalmologist. His anterior segments were normal, but fundi examination revealed dilated tortuous veins with scattered peripheral haemorrhages with Roth spots in both eyes. There was also bilateral mild macular oedema (image on the right above). The findings were in keeping with superficial retina ischaemia and impending central retinal vein occlusion (CRVO). Blood count showed a high WBC at 276 × 10^9^/L, Hb 121 g/L and platelets 458 × 10^9^/L and a blood film in keeping with CML. He had significant splenomegaly on examination. His *BCR‐ABL1* mutation screen was positive. Chronic‐phase CML was diagnosed and adequate effective treatment was started.

Ocular manifestations are more commonly described in acute leukaemia but have been described in 5–10% of patients with CML and have been linked to a poorer prognosis in patients with CML in some studies. Proliferative retinopathy is a feature of hyperleukocytosis, as seen in both patients. Other features of leukaemic retinopathy described include cotton wool spots, neovascularisation and perivascular sheathing. A delay in diagnosis can carry potentially severe consequences, including irreversible visual loss. A close collaboration between the general practitioners, ophthalmologists and haematologists is critical to ensuring prompt recognition and early management.

